# Comprehensive Analysis of Copy Number Variations on Glycoside Hydrolase 45 Genes among Different *Bursaphelenchus xylophilus* Strains

**DOI:** 10.3390/ijms232315323

**Published:** 2022-12-05

**Authors:** Xiaolei Ding, Ruiwen Zhao, Yonglin Dai, Yue Zhang, Sixi Lin, Jianren Ye

**Affiliations:** 1 Co-Innovation Center for Sustainable Forestry in Southern China, College of Forestry, Nanjing Forestry University, Nanjing 210037, China; 2 Jiangsu Key Laboratory for Prevention and Management of Invasive Species, Nanjing 210037, China

**Keywords:** *Bursaphelenchus xylophilus*, glycoside hydrolase 45, copy number variation, gene expression

## Abstract

*Bursaphelenchus xylophilus* is considered the most dangerous quarantine pest in China. It causes enormous economic and ecological losses in many countries from Asia and Europe. The glycoside hydrolase 45 gene family has been demonstrated in early studies to contribute to the cell wall degradation ability of *B. xylophilus* during its infection. However, the copy number variation (CNV) of the GH45 gene and its association with *B. xylophilus* pathogenicity were not fully elucidated. In this study, we found that the GH45 gene with two copies is the most predominant type among 259 *B. xylophilus* strains collected from China and Japan. Additionally, 18 strains are identified as GH45 genes with a single copy, and only two strains are verified to have three copies. Subsequent expression analysis and inoculation test suggest that the copy numbers of the GH45 gene are correlated with gene expression as well as the *B. xylophilus* pathogenicity. *B. xylophilus* strains with more copies of the GH45 gene usually exhibit more abundant expression and cause more severe wilt symptoms on pine trees. The aforementioned results indicated the potential regulatory effects of CNV in *B. xylophilus* and provided novel information to better understand the molecular pathogenesis of this devastating pest.

## 1. Introduction

*Bursaphelenchus xylophilus* is known as the causative agent of pine wilt disease, which originated in North America [1]. It normally does not harm local pine species in North America but causes enormous ecological and economic damage to all pine forests in Asia and Europe [2]. By the end of 2022, the infected areas of *B. xylophilus* will have reached over 700 counties in 19 provinces based on the annual announcement claimed by the State Forestry and Grassland Administration of China. In order to fully control this devastating disease, researchers have conducted various studies to elucidate the pathogenic mechanism of *B. xylophilus* [3,4,5]. With the help of the genome assembly of *B. xylophilus* [6,7], more and more genes, noncoding RNAs, and genomic variations were identified to expand our current knowledge of the molecular pathogenesis of this pest [8,9,10,11,12,13]. Among these, the glycoside hydrolase 45 (GH45) gene was reported to be expressed in the esophageal gland cells with cellulase digestion ability [14]. The subsequent study further indicates this gene family is likely to be acquired from bacteria via horizontal gene transfer and responsible for cell wall-degradation processes during infection [6,15]. This important gene family used to be considered a single copy in *B. xylophilus*, while our recent study proved that some of the GH45 genes had copy number variations (CNV) among different nematode strains [16].

Currently, relevant research on *B. xylophilus* usually focuses on identifying potential regulatory genes rather than structure variations like CNV [3,4,11]. However, it has been well-documented in many other organisms. Since 1980, researchers found that CNV in human genes was closely associated with gene expressions and further affected the development and progression of the human genomic disease. Subsequent studies indicated that CNV and other structural variations are widely distributed in the human genome and mostly occur within repeated genome regions like segmental duplications and tandem repeats [17,18,19]. Generally, CNV is considered a regulatory element to be responsible for gene diversity, gene expansion, and stress adaptation in humans, *Drosophila melanogaster*, and other model organisms [20,21,22,23]. Thus, it is necessary to explore the potential roles of GH45 CNV genes to shed light on the molecular mechanism of *B. xylophilus*. Despite their crucial biological importance, the accurate profiling of CNV genes is difficult due to their highly similar sequence contents [24]. Our preliminary study successfully found a unique primer pair to detect the CNV of a specific GH45 gene (located in the Contig002:2421000-2426000 region) and tested its validity in 4 nematode strains [16]. However, the aforementioned study did not provide a comprehensive landscape of such GH45 CNV genes among large *B. xylophilus* populations as well as their correlation with pathogenesis.

To fill this knowledge gap, we characterized the CNV of GH45 in 259 *B. xylophilus* strains to validate its variations with a large sample size. Additionally, the expression patterns of representative *B. xylophilus* strains with different GH45 copies were quantified. On top of that, the correlation between *B. xylophilu* CNV and pathogenicity was analyzed. We believe this more in-depth investigation of the GH45 gene with CNV by including sufficient nematode strains will provide us with comprehensive information to facilitate our understanding of the molecular pathogenesis of *B. xylophilus*.

## 2. Results

### 2.1. CNV of GH45 Gene Identified among Different B. xylophilus Strains

**Figure 1:**
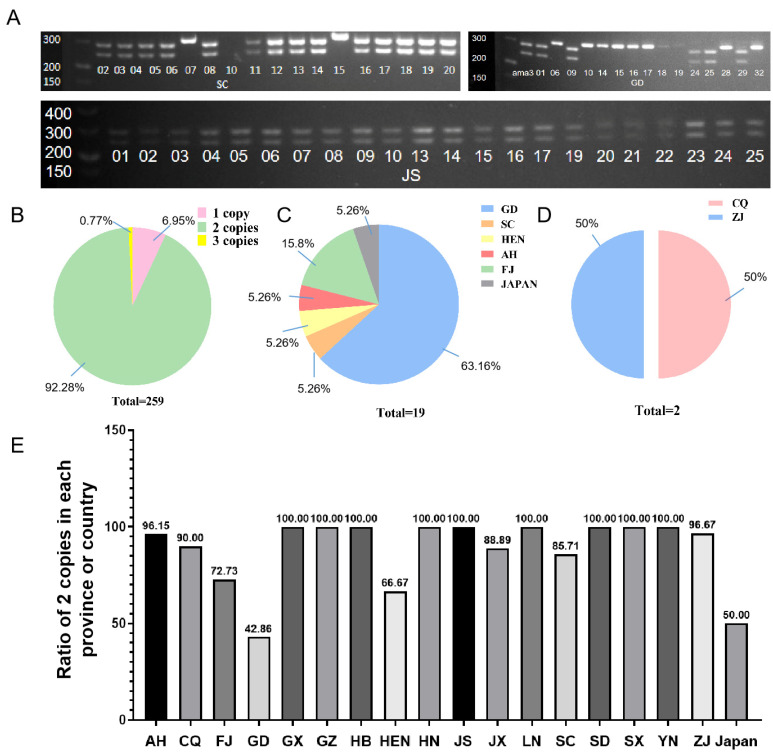
Distribution of copy number variations among different *B. xylophilus* strains. (**A**) Partial visualization result of CNV found in different nematode strains, SC means Sichuan province, GD means Guangdong, JS means Jiangsu, and the numbers indicate sample ID. (**B**) Percentage of different copies found in 259 *B. xylophilus* strains. (**C**) Geographical distributions of nematode strain with one copy, GD means Guangdong province, SC means Sichuan, HEN means Henan, AH means Anhui, and FJ means Fujian. (**D**) Geographical distributions of nematode strain with three copies, CQ means Chongqing, ZJ means Zhejiang. (**E**) Bar plot of the geographical distributions of *B. xylophilus* strains with two copies, AH means Anhui, CQ means Chongqing, FJ means Fujian, GD means Guangdong, GX means Guangxi, GZ means Guizhou, HB means Hubei, HEN means Henan, HN means Hunan, JS means Jiangsu, JX means Jiangxi, LN means Liaoning, SC means Sichuan, SD means Shandong, SX means Shaanxi, YN means Yunnan, ZJ means Zhejiang.

Generally, there are three different types of CNVs found in the GH45 gene among all 259 *B. xylophilus* strains based on PCR amplification. Strains with one DNA band indicate there is only one copy of GH45, while two and three bands indicate two and three copies, respectively (Figure 1A and Figure S1). Among all tested nematode strains, 92% (239) of all tested strains were identified to contain two copies of the GH45 gene (Figure 1B), 19 strains have one copy (Figure 1C), and two strains have three copies (Figure 1D). It is obvious that the CNV of the GH45 gene exists among *B. xylophilus* strains; meanwhile, the majority of *B. xylophilus* strains contain two copies. On the other hand, we found that all strains from Jiangsu, Hebei, Guangxi, Yunnan, Liaoning, Guizhou, Hunan, Shandong, and Shaanxi consistently had two copies (Figure 1E), while most strains (66.7%) from Guangdong province usually had only one copy (Figure 1B). The CNV of GH45 does not show significant differences among strains from the same province.

### 2.2. In Vitro Quantification of CNVs in GH45

**Figure 2:**
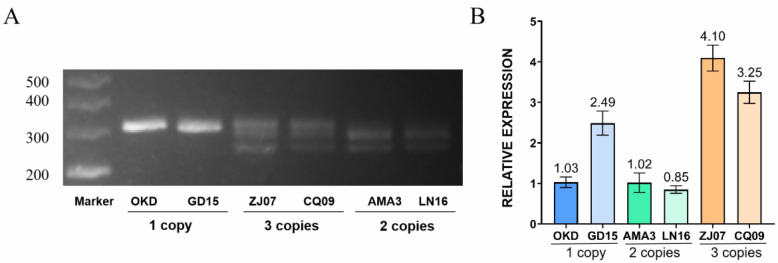
Gel visualization and in-vitro quantification of *B. xylophilus* strains with different copies. (**A**): CNV among tested strains visualized by 3% agarose gel. (**B**): Bar chart of expression level among 6 *B. xylophilus* using qPCR quantification.

Six Strains with different GH45 copy numbers and geographical origins (Supplementary material Table S1) were chosen for all downstream analysis (Figure 2A, OKD and GD15 have one copy; AMA3 and LN16 have two copies; CQ09 and ZJ07 have three copies). The quantification results suggested that strains with different copies exhibited different expression patterns when cultured with *Botrytis cinereal* shared by different copies of GH45 genes. ZJ07 showed the highest expression level among all tested strains, followed by CQ09. Additionally, all strains with less than three copies shared similar low expression levels except for GD15. Generally, the in-vitro expression of the GH45 gene among different *B. xylophilus* strains are correlated with their CNV. More copies would result in more abundant expression (Figure 2B).

### 2.3. Post-Inoculation Analysis of GH45 CNVs among Different B. xylophilus Strains

**Figure 3:**
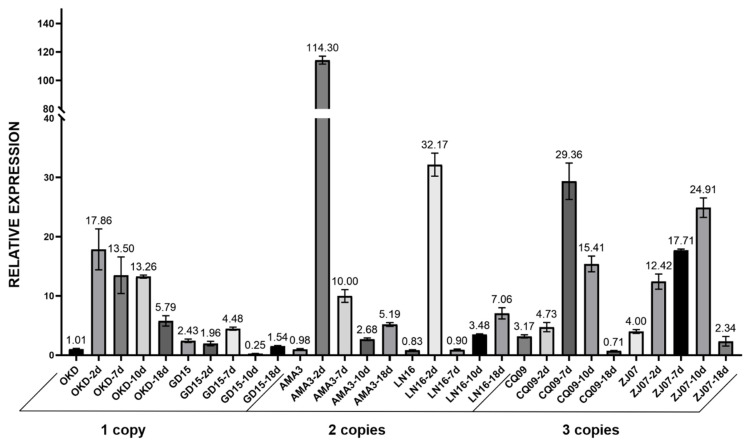
Relative expression of 6 *B. xylophilus* strains with different copy numbers after inoculating on pine trees for 2 days, 7 days, 10 days and 18 days.

The aforementioned 6 *B. xylophilus* strains were inoculated on *P. thunbergii* to detect their post-inoculation expression changes, respectively. Relative expression of each strain was quantified after inoculating for 2 days, 7 days, 10 days and 18 days. In general, the expression of the GH45 gene began to boost significantly after inoculation for 2 days and eventually declined after 18 days; meanwhile, strains with different copies exhibited different expression patterns (Figure 3). Strains with two GH45 copies showed the highest expression levels on day 2 but decreased sharply on the following days. Strains with one copy showed the average lowest expression levels and decreased eventually on day 18. Strains with three copies showed medium expression level and shared similar patterns with one copy strains.

**Figure 4:**
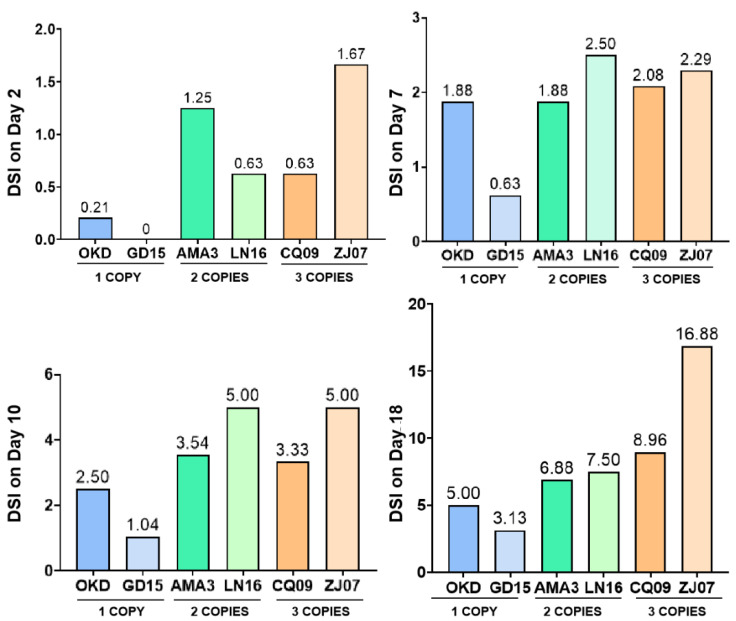
Disease severity index of different *B. xylophilus* strains.

The pathogenicity of the 6 *B. xylophilus* strains was also determined by calculating the disease severity index (DSI) at each sampling time. The DSI in the early stage (days 2, 7, and 10) did not show obvious differences among different strains since the symptoms were too mild to be classified (Figure 4). The DSI observed on day 18 suggested that strains with three copies of the GH45 gene could cause the most severe symptoms on pine trees, followed by strains with two copies, while the least severe symptoms were observed on strains with one copy (Figure 4). In combination with the expression and DSI results, we proposed that *B. xylophilus* strains with more copies of the GH45 gene would result in higher expression levels and induce more severe damage to the pine trees.

## 3. Discussion

Previous studies indicated GH45 gene families are obtained from fungi via HGT and are closely associated with the pathogenicity of *B. xylophlius* [6,15]. Our recent research verified that one of the GH45 genes possesses a CNV event with a limited sample size [16]. In this study, we further characterized the CNV of the GH45 gene using massive *B. xylophlius* strains to enhance our previous report. The profiling result indicates more than 90% of total strains in this study have two copies, 1.91% of total strains have one copy, and only two strains have three copies (Figure 1). It is proved that the gene with two copies is most common in *B. xylophlius* while one copy GH45 gene mainly exists in GD strains. Such distributions are somewhat consistent with our classification of *B. xylophlius* population structures based on SNPs (single nucleotide polymorphism) [16]. This population genetic study also suggested GD strains are very unique and showed a large genetic distance, with all other strains as well as a few strains from SC and HEN. Moreover, SNP based study demonstrated that most of the nematode strains shared very similar genomic variations as we found in this study. Theoretically, CNV can be potentially considered a novel biomarker in the population study of *B. xylophlius* if more genes with CNV can be found.

Expression analysis of *B. xylophlius* strains with different GH45 copies indicated that the overall expression levels began to increase in the early infection stage and decreased in the later stage. This observation indicated the GH45 genes are actively involved with the early infection activities when nematodes attempt to colonize pine trees and confront host defense [25,26]. Additionally, we found that *B. xylophilus* strains with two and three copies of the GH45 gene showed relatively higher expression levels than those with a single copy. It can be explained by the gene-dosage effect which was tagged by the associated CNV as reported in another relevant study [27]. For instance, some of the virus can adjust gene expression to deal with changing environments by modulating the copy number [28].

Although the influence of CNV on *B. xylophilus* pathogenicity was unknown, it has been widely studied in humans, animals, etc. [29,30,31,32,33,34]. Several researchers suggested that pathogenicity-associated genes or regulator genes with more copies would cause more severe disease symptoms [35,36]. This assumption also corresponds to the result of our study in which *B. xylophilus* strains with the most abundant GH45 copies showed the strongest pathogenicity (Figure 4). Meanwhile, the different copies of the GH45 gene varied mostly on the number of tandem repeats that occurred within the ORF region, as reported in our previous study [16]. Thus, the more copy of the GH45 gene will also diversify its corresponding protein product to reinforce further the infection ability of *B. xylophilus*. Consequently, we believe the CNV found in the GH45 gene plays crucial role in the pathogenesis of pine wilt disease. The infection capacity of *B. xylophilus* could be enhanced by introducing more CNV to alter individual gene expression and increase isoform diversity.

Normally, CNV represents regions of the genome that are duplicated or deleted in different individuals [37]. Based on our study, it is likely that some of the *B. xylophilus* pathogenicity-associated genes are prone to have CNV. The occurrence of CNV will regulate and alter the functions of such genes by changing the overall expressions and protein sequences. It is necessary to focus on other pathogenicity-associated genes to verify the existence of CNV. Since it has been demonstrated that the dosage sensitivity of individual genes is a common cause of CNV pathogenicity in humans and mammals [38,39]. Such studies have pointed out that the dosage sensitivity of CNVs can influence pathogenicity.

## 4. Materials and Methods

### 4.1. Collection of Bursaphelenchus xylophilus Strains

A total of 259 *B. xylophilus* strains (Supplementary Table S1) used in this study were collected from different areas in China, together with 2 strains from Japan [16]. OKD (Japan), GD15 (Guangdong, China), AMA3 (Anhui, China), LN16 (Liaoning, China), CQ09 (Chongqing, China), and ZJ07 (Zhejiang, China) were selected to perform inoculation test based on CNV identification. All aforementioned *B. xylophilus* strains were cultured on *Botrytis cinerea* for 7 days under 28 °C to obtain adequate samples for downstream experiments. The *Botrytis cinerea* was stored in the laboratory of Prof. Jianren Ye at Nanjing Forestry University. The fungus was cut into 6 mm diameter blocks with sterilized perforator and cultured on PDA media for 5–7 days in a dark incubator at 25 °C.

According to the Baermann funnel method, nematodes were isolated from culture media and stored in sterilized water using 1.5 mL centrifuge tubes. After washing three times in sterilized water, the *B. xylophilus* was concentrated by centrifugation at 1500× *g* for 3 min and frozen in liquid nitrogen for subsequent experiments. The nematodes were collected from infected wood and stored in 15 mL centrifuge tubes with sterilized water. Using an Eppendorf pipette gun to extract water with nematodes, then placed on a glass slide. The microscopic identification was carried out by a Zeiss microscope (Leica DM500) and complied with its morphological characters [40,41]. The molecular detection was conducted with a detection kit developed by ourselves. After extracting the DNA of examined nematodes, qRT-PCR based on Taqman method was conducted. If the Ct value is under 30, then the nematode will be identified as *Bursaphelenchus xylophilus* [42].

### 4.2. Identification of CNV on Glycoside Hydrolase 45

The genomic DNA was extracted based on CTAB methods [43]. Specific primers for CNV identification were identified in our early study based on tandem repeat variations that occurred among different copies of GH45 genes (sense primer: CGTGGTGGAAGGTAAGAC, antisense primer: GTTGGAGTGGTGCTTGAT) [16]. PCR was conducted using the following procedure: 94 °C for 2 min, followed by 35 cycles (94 °C for 30 s, 58 °C for 30 s, and 72 °C for 30 s), and 72 °C for 10 min. The exact copy numbers can be assessed based on fragment lengths by visualizing the PCR products with 3% agarose gel electrophoresis.

### 4.3. Inoculation Test and Disease Severity Analysis

From OKD, GD15, AMA3, LN16, CQ09, and ZJ07 strains, 3000 *B. xylophilus* individuals were inoculated into a small wound of two-year-old *Pinus thunbergii* stems for 2 days, 7 days, 10 days, and 18 days, and cultivated at temperatures from 28 to 35 °C, respectively. The inoculation test was implemented from 14 July to 1 August, with three replicates for each sampling time. The *B. xylophilus* were isolated from the cut stems for 6 h, using the Baermann funnel method. The disease level was recorded as follows: level 0, healthy; level 1, under 1/2 of needles chlorisis, under 1/4 of needles yellowing; level 2, above 1/2 of needles chlorisis, above 1/4 of needles yellowing; level 3, 3/4 of needles yellowing, under 1/2 of needles reddening; level 4, above 1/2 of needles reddening, pine trees dying or dead. The disease severity index (*I*_D_) was calculated according to the following formula: *I*_D_ = Σ(x_i_a_i_)/Σx_i_ × a_max_ × 100. In this formula, x_i_ means the number of trees at the same level, a_i_ means the disease level, a_max_ represents the highest disease level [44,45,46]. Afterward, the disease severity index (DSI) of different strains was collected and visualized by GraphpadPrism 8.0.1.

### 4.4. Quantification of GH45 Gene with Different Copies

Total RNAs of *B. xylophilus* strains used for the inoculation test were extracted using Trizol reagent (Yuanye, Shanghai, China) and assessed by Nanodrop 2000 (http://www.nanodrop.com/Productnd2000overview.aspx, accessed on 1 October 2022) [47]. Qualified RNAs were synthesized into cDNAs using PrimeScript™ RT reagent Kit (TAKARA, Dalian, China) for qPCR assay. Specific primers for qPCR assays were selected from the conserved region shared by different copies of GH45 genes (sense primer: CGACCATGAATAAACTACTCGTTTC, antisense primer: TAGATGGTGCTCCACTGGC). The quantitative PCR was conducted by an ABI 7900HT-Sequence Detection System (Applied Biosystems, Carlsbad, CA, USA), with TB Green^®^ Premix Ex Taq™ (Ti RNaseH Plus), ROX plus kit (TAKARA, Dalian, China) with three replicates.

## 5. Conclusions

The comprehensive landscape of GH45 CNV is well characterized in this study. We find that GH45 with two copies is the most prevalent type among all 259 *B. xylophilus* strains. Furthermore, *B. xylophilus* strains with multiple copies show higher expression levels and pathogenicity than those with a single copy. In general, this study demonstrates the wide existence of structure variants like CNV in *B. xylophilus* and illuminates the potential regulatory roles of CNV on the pathogenicity of *B. xylophilus*.

## Data Availability

Strains and primers are available upon request. The authors affirm that all data necessary for confirming the conclusions of the article are present within the article, figures, and tables.

## Supplementary Materials

The following are available online at https://www.mdpi.com/article/10.3390/ijms232315323/s1.
