# Supplementary material for: Comprehensive Analysis of Copy Number Variations on Glycoside Hydrolase 45 Genes among Different Bursaphelenchus xylophilus Strains
Source: Int J Mol Sci. 2022 Dec 5;23(23):15323. doi: 10.3390/ijms232315323 (PMC9735991; doi:10.3390/ijms232315323)
Supplement: Supplementary file 1 [file ijms-23-15323-s001.zip › Supplementary Figure S1.pdf]

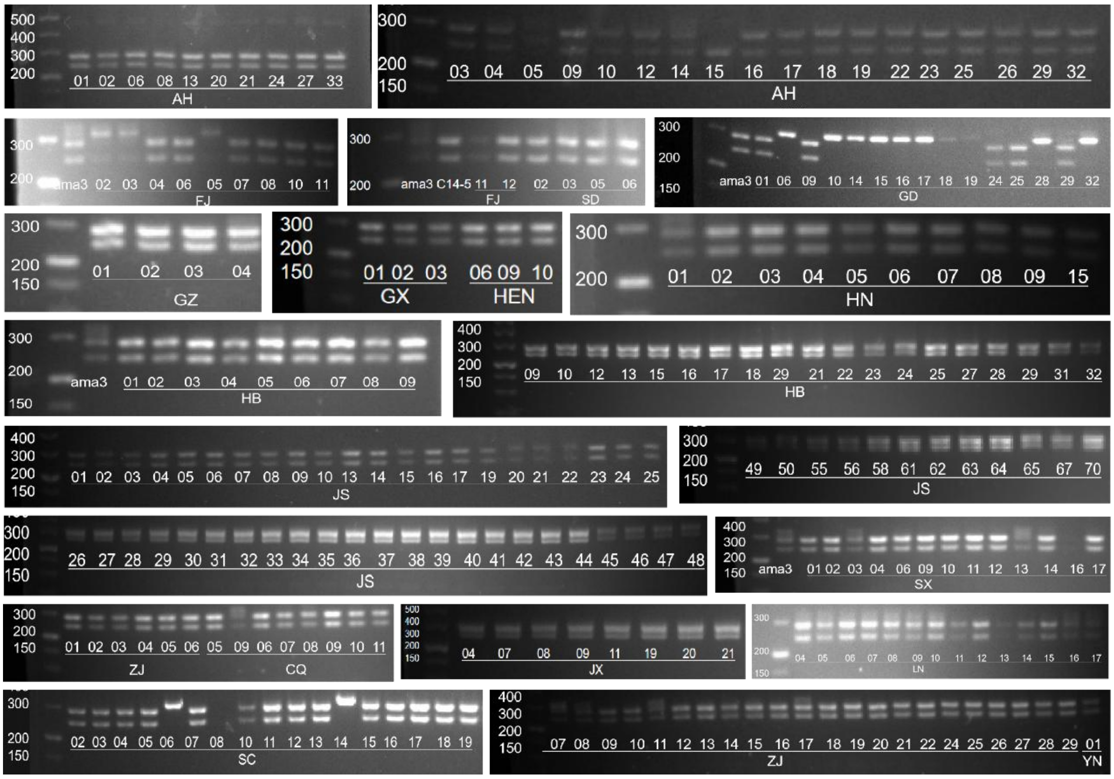

Supplementary Figure S1. Visualization result of GH45 CNV found in different *B. xylophilus* strains. AH means Anhui, CQ means Chongqing, FJ means Fujian, GD means Guangdong, GX means Guangxi, GZ means Guizhou, HB means Hubei, HEN means Henan, HN means Hunan, JS means Jiangsu, JX means Jiangxi, LN means Liaoning, SC means Sichuan, SD means Shandong, SX means Shaanxi, YN means Yunnan, ZJ means Zhejiang, the figures mean the strain number.
